# Botulinum toxin type A alleviates neuropathic pain and suppresses inflammatory cytokines release from microglia by targeting TLR2/MyD88 and SNAP23

**DOI:** 10.1186/s13578-020-00501-4

**Published:** 2020-12-09

**Authors:** Xuan Wang, Sheng Tian, Hansen Wang, Pan Liu, Heqing Zheng, Lanxiang Wu, Qian Liu, Wei Wu

**Affiliations:** 1 Department of Neurology, The Second Affiliated Hospital of Nanchang University, No.1 Minde Road, NanchangJiangxi Province, 330006 China; 2 Imaging Department, The Second Affiliated Hospital of Nanchang University, NanchangJiangxi Province, 330006 China

**Keywords:** Neuropathic pain, Botulinum toxin type A, Microglia, Synaptosome associated protein 23

## Abstract

**Background:**

Botulinum toxin type A (BTX-A) was considered to be a new potential drug for neuropathic pain (NP) treatment.

**Results:**

In vivo, BTX-A attenuated chronic compression injury (CCI)-induced pain in rats, and reduced production of pro-inflammatory factors. The inhibition of BTX-A to expression and phosphorylation of SNAP23 were partly reversed by TLR2/MyD88 upregulation. In LPS-stimulated microglia, we also found that BTX-A suppressed TLR2, MyD88, p-SNAP23 and SNAP23 expression, and reduced pro-inflammatory factors secretion. Upregulation of TLR2 and MyD88 recued the inhibition of BTX-A to LPS-induced activation of SNAP23. Then, we demonstrated that BTX-A reduced expression of SNAP23 through inhibition of IKKα/β phosphorylation. Besides, the inhibition of BTX-A to LPS-induced upregulation of SNAP23 can be reversed by proteasome inhibitor. NEDD4, an E3 ubiquitin ligase, was proved to be bind with SNAP23. BTX-A reduced expression of SNAP23 via facilitating ubiquitin-mediated degradation of SNAP23.

**Conclusion:**

Overall, our data demonstrated that BTX-A attenuated NP via reducing the secretion of pro-inflammatory factors from microglia by inhibition of TLR2/MyD88 signaling. BTX-A downregulated expression of SNAP23 via reducing phosphorylation of IKKα/β, and enhancing ubiquitination of SNAP23 by suppressing TLR2/MyD88 signaling.

## Background

Neuropathic pain (NP) is a pain syndrome characterized by the spontaneously intermittent or ongoing burning pain, allodynia and hyperalgesia. NP resulted from the nervous system damage induced by trauma (such as brain injury and spinal) and multiple disorders (such as malignant tumors, stroke and diabetics) [1]. According to the research of epidemiological, NP affects approximately 6.9–10% population. Medical expense of NP is increasing year by year. NP becomes a major public health problem in the world, and a global burden of medical systems [2]. Nevertheless, NP usually companied with an unsatisfactory treatment outcome due to partly drugs not suitable to europathic pain, and in primary care, the contribution of neuropathy to pain was unrecognized [3].

At present, numerous new compounds for NP treatment are in preclinical development. For example, serotonin modulators, vanilloid receptor antagonists and inhibitor of apoptosis [4]. Interestingly, some recent studies pointed out botulinum toxin type A (BTX-A), an older drug for muscular hyperactivity-associated disease and cosmetic surgery treatment, is a new potential strategy in NP therapy [5]. It was reported that BTX-A could cleave the synaptosome associated protein 25 (SNAP25) at different soluble N-ethylmaleimide sensitive factor attachment protein receptor motifs, thus to inhibit the release of neurotransmitter [6]. Our previous study also demonstrated that BTX-A could effectively improve chronic compression injury (CCI)-induced NP in SD rats. Meanwhile, we found that BTX-A plays its effect through targeting microglia [7]. Hence, we further explored the action mechanism of BTX-A in NP in this present study.

BTX-A belongs to BTX protein group, produced by *Clostridium botulinum*, that is an anaerobic bacteria [8]. Reliable evidences proved that BTX-A attenuates NP through targeting toll-like receptor 2 (TLR2). In addition, BTX-A affects SNAP23 and SNAP25 expression in neuronal cells, inhibits production of pro-inflammatory factors (IL-1β, IL-18 and IL-6) and expression of SNAP23 via targeting TLR2 and MyD88, an adaptor protein of TLR2, in lipopolysaccharide (LPS)-stimulated microglia [9, 10]. However, the definite mechanism of action of BTX-A improves NP through targeting TLR2/MyD88 signaling remains unclear. It was indicated that MyD88-dependent TLR signaling regulates phosphorylation of SNAP23 [11]. These studies revealed that BTX-A maybe reduce expression of SNAP23 by epigenetic modification.

In this present study, we proved that BTX-A attenuated CCI-induced neuropathic pain in rats, reduced releasing of pro-inflammatory factors from activated microglia and expression of SNAP23 through inhibition TLR2 expression. Mechanismly, BTX-A inhibited expression of SNAP23 by two epigenetic modifications. On the one hand, BTX-A suppressed expression and phosphorylation of SNAP23 via inhibition IκB kinase α (IKKα)-IKKβ complex (IKKα/β) phosphorylation. On the other hand, BTX-A promoted ubiquitination of SNAP23 via facilitating expression of neural precursor cell-expressed developmentally downregulated gene 4 (NEDD4). Overall, our data revealed a new regulatory mechanism of BTX-A in treatment of NP.

## Materials and methods

### Animals

Specific pathogen-free (SPF) Sprague–Dawley (SD) rats (gender, male, weight, 200–250 g) were purchased from Charles River (Beijing, China). All animal experiments were fulfilled strictly according to the guidelines for the Care and Use of Laboratory Animals of the National Institutes of Health, and were approved by the Animal Care and Use Committee of The Second Affiliated Hospital of Nanchang University. All animals were raised in a comfortable environment with 22 ± 2 °C temperature and a 12 h light–dark cycle. Meanwhile, the animals were given enough food and water.

### Chronic constriction injury rat model

For the NP rat model, animals were accepted with CCI operation as our previous study [7]. After three days for the operation, NP rats were randomly treated with saline, 10 U/kg BTX-A (Allergan Pharaceuticals Ireland) or 20 U/kg BTX-A through subcutaneous injection into metatarsal surface. In addition, the rats in sham-operation group were injected with equal saline. Animals were divided into four groups: Sham, NP, BTX-A-10 and BTX-A-20. Next, at 14 days later for CCI operation, spinal cord tissues from L4-L6 segment were stripped. Subsequently, the expression of TLR2, TLR4 and MyD88 in gene and protein levels were detected using qRT-PCR and western blotting assay, respectively.

### QRT-PCR assay

TRIzol reagent (Invitrogen, Carlsbad, CA, USA) was used to isolate total RNA from tissues and cells according to the introduction. Then, 400 ng of total RNA were converted into complementary DNA using a PrimeScript RT Reagent Kit (TaKaRa, Tokyo, Japan). Next, the relative expression of genes were determined using SYBR Premix Dimmer Eraser kit (TaKaRa) on a Thermal Cycler Dice Real Time System (ABI7900 system, Applied Biosystems, Foster City, CA, USA). Finally, the relative quantification of genes were calculated using the 2^−ΔΔCt^ method. GAPDH was served as the loading control.

### Western blotting assay

RIPA lysis buffer (Sigma-Aldrich, St. Louis, MO, USA) was utilized to isolate total protein from tissues and cells. Next, the concentration of total protein were ensured using a NanoDrop 2000 spectrophotometer (Thermo Scientific, USA). Equal 20 μg of protein was loaded and then separated on a 10% SDS-PAGE gel. After that, all proteins were transferred into PVDF membranes (Millipore Corp., Billerica, MA, USA). Subsequently, the membranes were incubated with 5% non-fat mike for 1 h at room temperature. All membranes were maintained with the working solution of primary antibodies against TLR2 (1:1000, Abcam, Cambridge, MA, USA), TLR4 (1:1000, Abcam), MyD88 (1:1000, Abcam), p-SNAP23 (1:2000, Cell Signaling Technology, Danvers, MA, USA), SNAP23 (1:1000, Cell Signaling Technology), p-IKKα/β (1:2000, Cell Signaling Technology), NEDD4 (1:1000, Abcam), HA (1:2000, Abcam) and β-actin (1:1000; Santa Cruz Biotechnology, Dallas, TX, USA) at 4 °C for overnight. Then, all membranes were maintained with HRP-conjugated secondary goat anti-rabbit or anti-mouse antibody (Beijing TransGen Biotech Co., Ltd., Beijing, China). Finally, the protein bands were visualized using an ECL kit (Applygen Technologies Inc., Beijing, China) and subsequent analyzed using the software of Image-Pro Plus 6.0.

### Pain threshold determination

To clarify whether BTX-A attenuates neuropathic pain in NP rats via inhibition of TLR2, all rats were randomly divided into five groups: sham, NP, BTX-A, BTX-A + LV-NC and BTX-A + LV-TLR2. Overexpression plasmid of TLR2 (pcDNA-TLR2) was constructed, and then were packaged into lentivirus obtained from GenePharma Corporation (Shanghai, China). In sham and NP group, the rats were given with saline at 3 days later for CCI treatment. In BTX-A group, the rats were given with 20 U/kg BTX-A at 3 days later for CCI treatment. In BTX-A + LV-NC group, the rats were injected with 20 U/kg of BTX-A and 5 μl of lentivirus carrying empty vector (LV-NC, 1 × 10^9^ TU/ml). In BTX-A + LV-TLR2, the rats were injected with 20 U/kg of BTX-A and 5 μl of lentivirus carrying pcDNA-TLR2 (LV-TLR2, 1 × 10^9^ TU/ml). The lentivirus were injected into rats through tail intravenous. At 0, 3, 5, 7, 9, 11, 13, 15 days post-CCI, the mechanical withdrawal threshold (MWT) and thermal withdrawal latency (TWL) of each rat were examined as our previous study [7].

### Immunohistochemistry assay

Immunohistochemistry assay was carried out to assess the expression of Iba1, a marker of activated microglia, in spinal cord tissues. After 14 days for CCI operation, spinal cord segments were obtained from each rat, and were then fixed with 4% paraformaldehyde. Tissues followed by cut into consecutive slices with 4 μm thick using a microtomes. Following dewaxed and rehydrated, sections were incubated with citrate buffer for antigen retrieval. Next, sections were blocked with 2% bovine serum albumin, and then primary antibody against Iba1 (1:1000, Abcam) at 4 °C for overnight. After that, sections were incubated with the HRP-labeled secondary antibody (1:1000, Abcam) for 30 min at 37 °C. Then, sections were stained with DAB working solution (Solarbio life science, Beijing, China) for 5 min in the dark. Finally, expression of Iba1 was analyzed using a microscope.

### ELISA assay

The concentration of pro-inflammatory factors (IL-1β, IL-6 and TNF-α) in serum and cell supernatant were ensured by ELISA assay. The specific ELISA kits were obtained from R&D Systems (Minneapolis, MN, USA), and all detections were performed according to the manufacture’s protocol.

### Microglia culture and treatment

Rat microglia cell line HAPI was purchased from BeNa Culture Collection (Suzhou, China), and were cultured in the high glucose DMEM medium (Gibco, Thermo Fisher Scientific Inc., Waltham, MA, USA) supplemented with 10% fetal bovine serum (Gibco) in a humidified incubator with 5% CO_2_ at 37 °C. Then, 100 ng/ml of lipopolysaccharide (LPS; Sigma-Aldrich), 100 nM of BTX-A, 100 μg/ml of cycloheximide (CHX, Sigma-Aldrich) and 15 μM of MG132 (Sigma) were used to stimulate microglia for different experiments.

### Cell transfection

NEDD4 siRNA, MDM2 siRNA, and the scramble siRNA were obtained from Sangon Biotech (Shanghai, China). Full-length sequences of TLR2, MyD88, SNAP23, NEDD4 and TAK1 were sub-cloned into pcDNA3.1 vector (Invitrogen), respectively. Overexpression system of TLR2 (pcDNA-TLR2), MyD88 (pcDNA-MyD88), SNAP23 (pcDNA-SNAP23), NEDD4 (pcDNA-NEDD4) and TAK1 (pcDNA-TAK1) were obtained. CMV promoter was selected for high-level gene expression. These siRNAs, plasmids and the empty vector were transfected into microglia using Lipofectamine 2000™ reagent (Invitrogen) according to the specific introduction. At 48 h later for transfection, following experiments were done.

### Co-immunoprecipitation (Co-IP) assay

At 24 h later for LPD and BTX-A treatment, microglias were harvested with RIPA lysis buffer. Then, the immunoprecipitation was carried out with anti-NEDD4. Cell lysates containing 2 mg total protein were incubated with anti-NEDD4/protein A agarose (sc2001, Santa Cruz) complex for overnight at 4 °C. Then, the breads were washed with TBST buffer for trice, and 4 × SDS-PAGE sample buffer for one time. Finally, proteins were separated on a 10% SDS-PAGE gel. The interaction between NEDD4 and SNAP23 was ensured using western blotting assay by anti-SNAP23.

### SNAP23 ubiquitination detection

To explore whether NDEE4 results the ubiquitination of SNAP23, pcDNA-NEDD4 and HA-labeled Ubiquitin (HA-Ub) were co-transfected into HEK293T cells using Lipofectamine 2000™ reagent. At 48 h later, cells were maintained with 15 μM MG132 for 2 h. Finally, western blotting assay was performed by anti-SNAP23 and anti-HA. Level of SNAP23 ubiquitination was analyzed according to the expression of SNAP23 and HA-Ub.

### Statistical analysis

SPSS version 20.0 (IBM, Chicago, IL, USA) was used to accomplish the statistical analyses, and the data were presented as the mean ± standard deviation (SD). For the analysis between two independent groups, Student’s *t*-test was performed. One-way analysis of variance was used to analyze differences among multiple groups. A value of *P* less than 0.05 was recognized as statistically significant.

## Results

### BTX-A inhibited TLR2 and MyD88 expression in NP rats

**Fig. 1:**
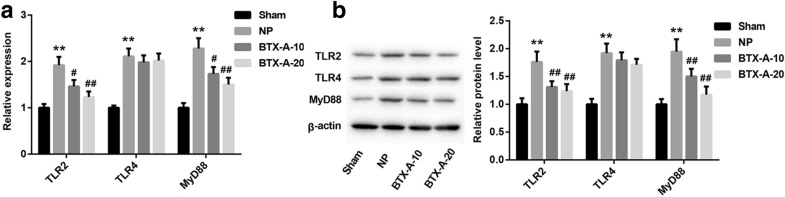
Expression of TLR2 and MyD88 were suppressed by BTX-A. SD rats were randomly divided into four groups: Sham, CCI-induced NP, BTX-A-10 (rats were given 10 U/kg of BTX-A following CCI) and BTX-A-20 (rats were given 20 U/kg of BTX-A following CCI). **a** Expression of TLR2, TLR4 and MyD88 mRNAs were measured by qRT-PCR. **b** Expression of TLR2, TLR4 and MyD88 proteins were examined using western blotting assay. **P < 0.01 compared with Sham group, ^#^*P* < 0.05 and ^##^*P* < 0.01 compared with NP group

It was reported that the expression of TLR2 in LPS-stimulated microglia was proved to be affect by BTX-A, while BTX-A has no effect on the expression of TLR4 [9]. Here, we explored the effect of BTX-A on expression of TLR2, TLR4 and MyD88 in NP rats. Our data showed that the expression of *TLR2*, *TLR4* and *MyD88* were upregulated in spinal cord tissues from NP rats. We also found that the expression of *TLR2* and *MyD88* were decreased in NP rats by BTX-A treatment (Fig. 1a). Western blotting assay indicated that BTX-A could significantly inhibit the highly expressed TLR2 protein and MyD88 protein in NP rats (Fig. 1b). Overall, BTX-A could suppress the expression of TLR2 and MyD88 in NP rats, but has no effect on the expression of TLR4.

### BTX-A alleviated pain in NP rats through inhibition of TLR2

**Fig. 2:**
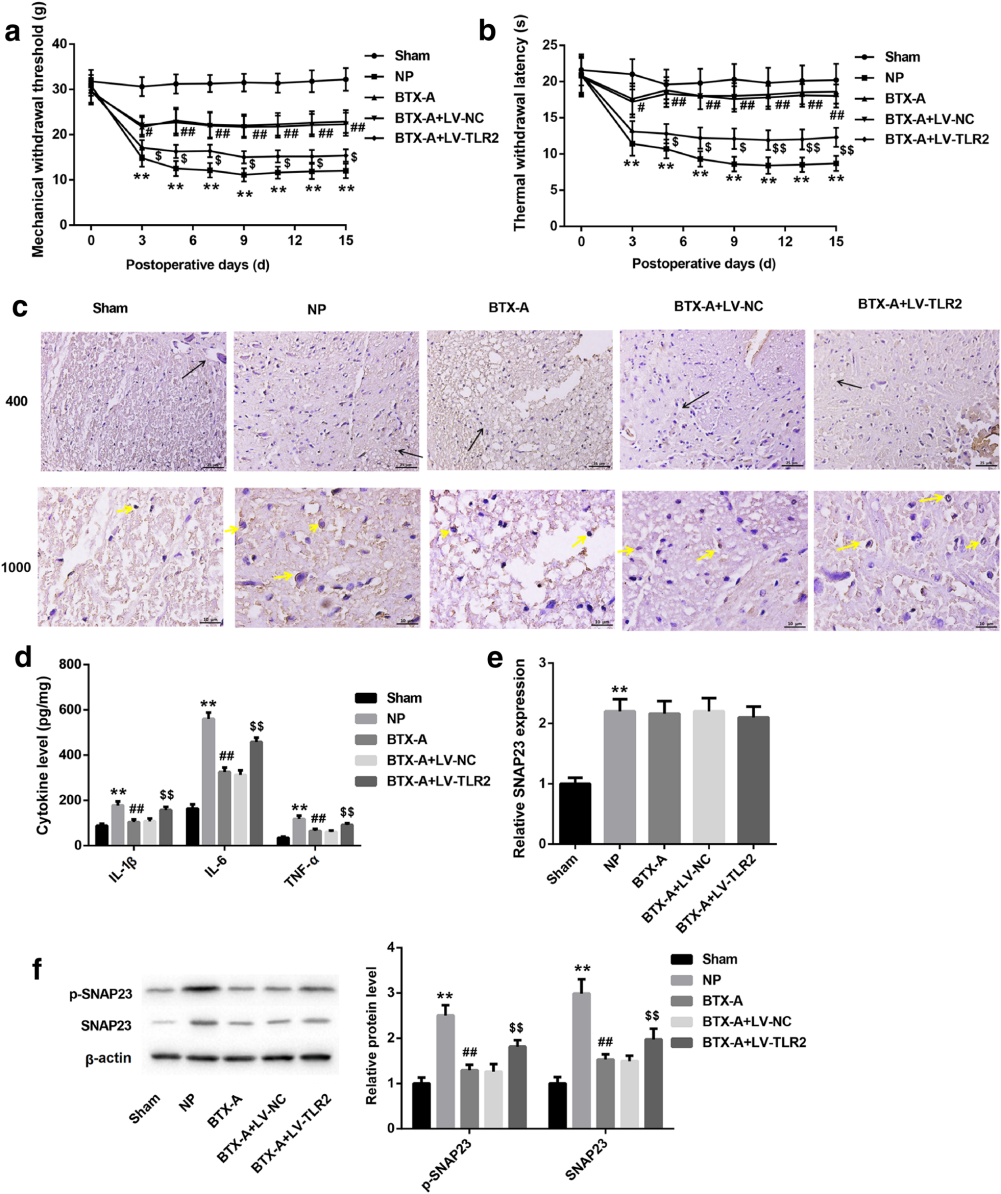
BTX-A elevated pain threshold of NP rats through targeting TLR2. SD rats were randomly divided into five groups: Sham, CCI-induced NP, BTX-A (rats were given 20 U/kg of BTX-A following CCI), BTX-A + LV-NC (NP rats were given 20 U/kg of BTX-A and lentivirus carrying NC) and BTX-A + LV-TLR2 (NP rats were given 20 U/kg of BTX-A and lentivirus carrying pcDNA-TLR2). **a** Threshold of mechanical withdrawal in rats were measured at 0, 3, 5, 7, 9, 11, 13, 15 days after induction of CCI. **b** Latency of thermal withdrawal in rats also were ensured at 0, 3, 5, 7, 9, 11, 13, 15 days after induction of CCI. Blank arrows indicate the boundary between gray matter and white matter. Yellow arrows indicate the glia cells. **c** Expression of Iba1 in spinal cord tissues was detected using immunohistochemistry assay. **d** Concentration of IL-1β, IL-6 and TNF-α were examined using ELISA. **e** qRT-PCR was performed to detect expression of SNAP23 mRNA. **f** Western blotting assay was carried out to examine levels of p-SNAP23 and SNAP23 in spinal cord tissues. **P < 0.01 contrasted with Sham group, ^##^*P* < 0.01 contrasted with NP group, and ^$$^*P* < 0.01 compared with BTX-A group

To explore whether BTX-A improves neuropathic pain in NP rats via targeting TLR2, we constructed the overexpression system of TLR2 using lentivirus. BTX-A at a dose of 20 U/kg was used to treat NP rats infected with or with LV-TLR2. Lentivirus successfully infected the spinal cord tissues of rats (Additional file 1: Figure S1). Our results proved that the MWT and TWL in NP rats notably lower than normal rats. BTX-A treatment obviously upregulated the MWT and TWL in NP rats, but the effect of BTX-A were partly reversed by TLR2 (Fig. 2a and b). Our data showed that BTX-A elevated pain threshold in rats post-CCI through inhibition of TLR2 expression. Subsequently, we detected the expression of Iba1, a marker of activated microglia, in spinal cord tissues of rats. Here, the immunohistochemistry result proved that CCI-induced activation of microglia in NP rats can be repressed by BTX-A treatment. However, the inhibitory effect of BTX-A was notably reversed by TLR2 (Fig. 2c). Moreover, we measured the concentration of pro-inflammatory factors (IL-1β, IL-6 and TNF-α) in peripheral blood of rats. As shown in Fig. 2d, the production of these cytokines were upregulated in NP rats, and were then inhibited by BTX-A treatment. We further found that the expression of SNAP23 mRNA in NP rats was increased. However, BTX-A together with or without LV-TLR2 have no effect on the expression of SNAP23 mRNA (Fig. 2e). Interestingly, BTX-A effectively suppressed the expression of SNAP23 in protein level and the phosphorylation of SNAP23 via inhibition of TLR2 (Fig. 2f). Overall, in vivo experiments, we found that BTX-A relieved the pain in NP rats via impeding the activation of microglia, the expression of SNAP23, and the phosphorylation of SNAP23 by inhibition of TLR2 expression.

### BTX-A inhibited the releasing of inflammatory factors through suppression of TLR2/MyD88/SNAP23 signaling

**Fig. 3:**
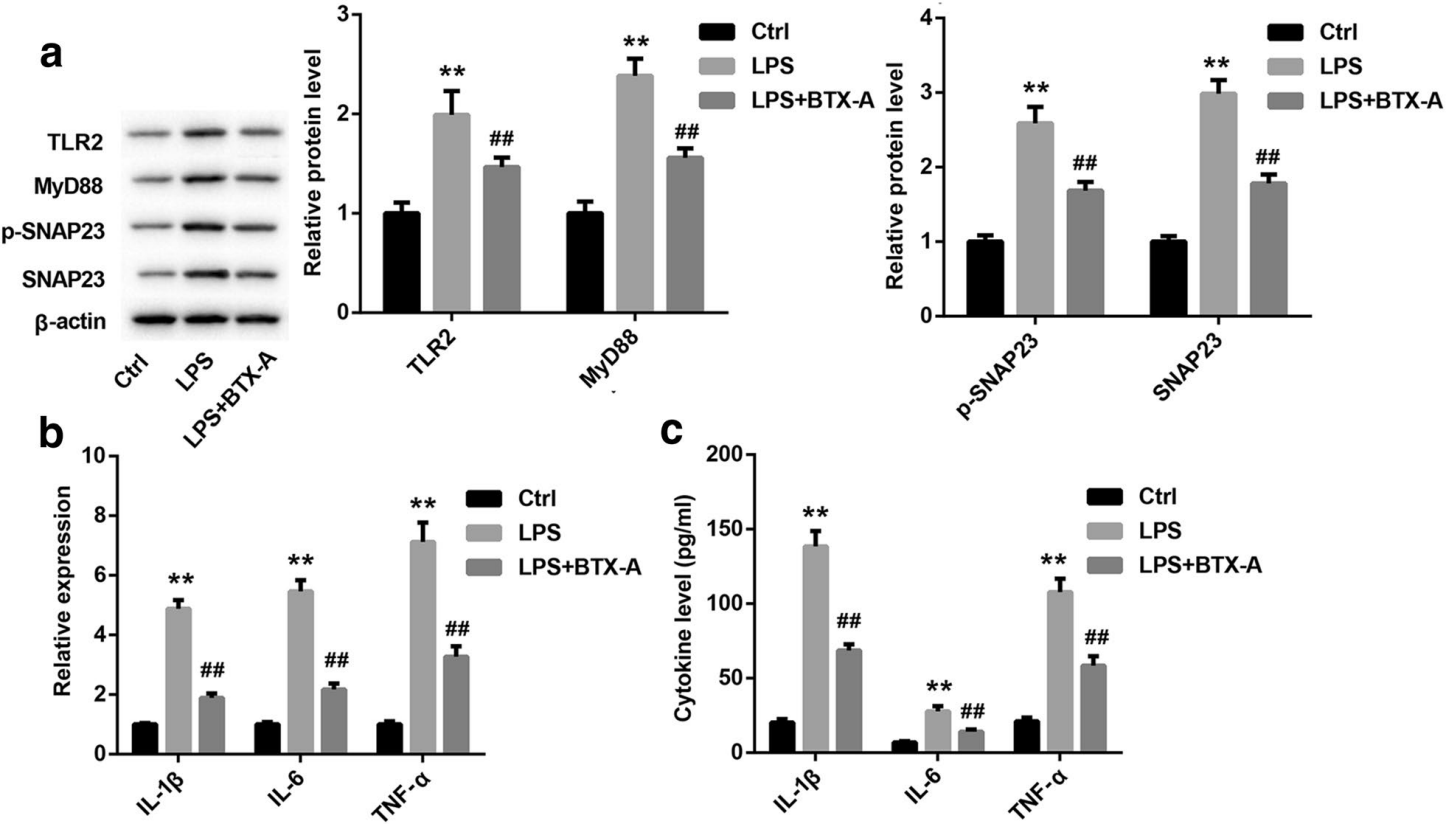
BTX-A restrained the effect of LPS on TLR2, MyD88, SNAP23 and pro-inflammatory factors expression. Microglias were co-treated with LPB and BTX-A. **a** Expression of TLR2, MyD88, p-SNAP23 and SNAP23 were measured by western blotting assay. **b** Expression of IL-1β, IL-6 and TNF-α genes were examined using qRT-PCR. **c** ELISA assay was performed to measure the concentration of IL-1β, IL-6 and TNF-α in cell culture medium. **P < 0.01 contrasted with Ctrl group and ^##^*P* < 0.01 contrasted with LPS group. The cells in Ctrl group was treated with nothing

To investigate the regulatory mechanism of BTX-A on SNAP23 expression and phosphorylation and releasing of pro-inflammatory factors from microglia, we used LPS to active microglia. Our data showed that LPS significantly facilitated the expression of TLR2, MyD88, p-SNAP23 and SNAP23, while the promotory effect of LPS was inhibited by BTX-A (Fig. 3a). LPS-induced the upregulation of IL-1β, IL-6 and TNF-α mRNAs in microglia were partly reversed by BTX-A (Fig. 3b). In addition, BTX-A markedly repressed the production of IL-1β, IL-6 and TNF-α in LPS-treated microglia (Fig. 3c).

**Fig. 4:**
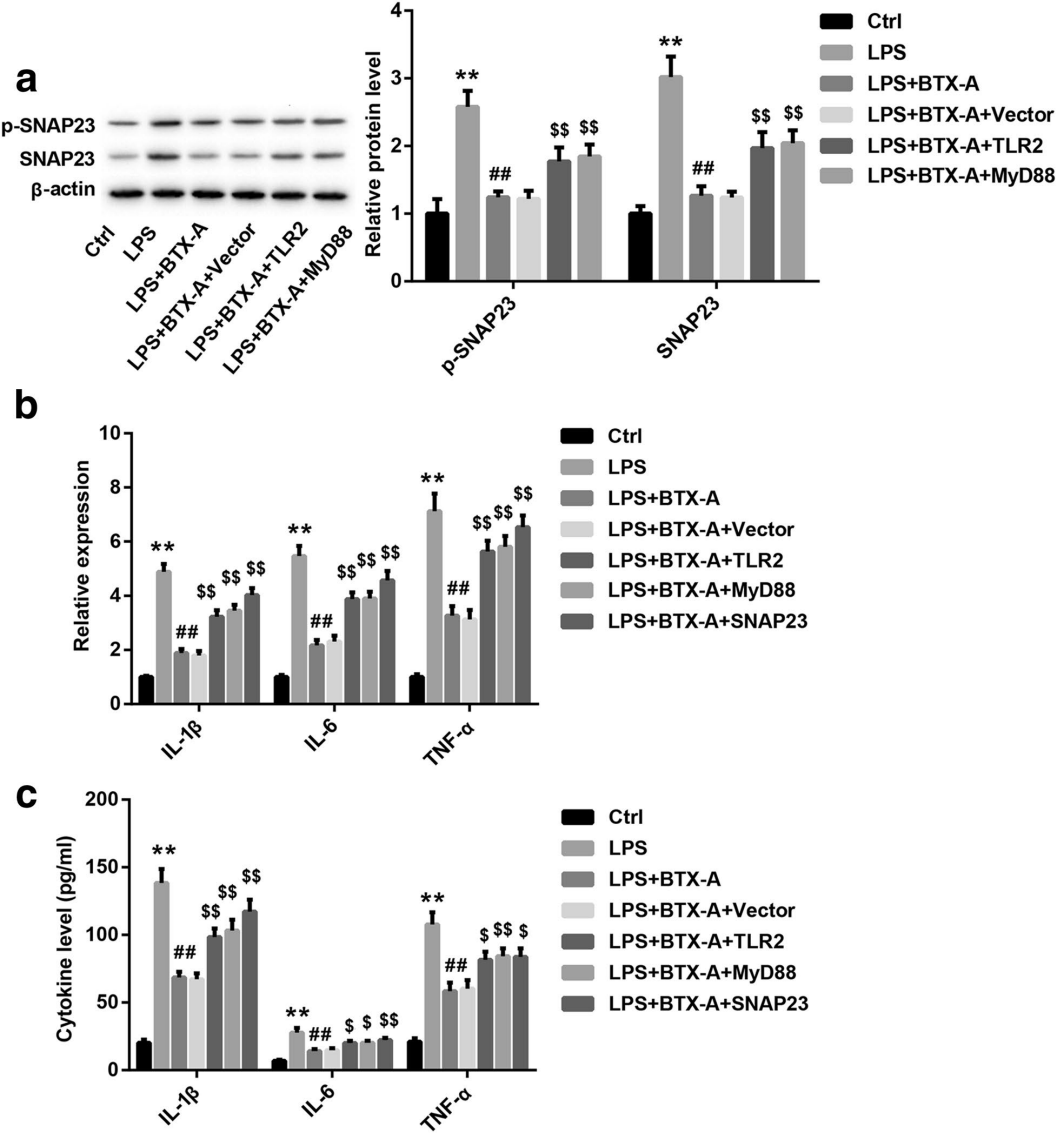
BTX-A repressed LPS-induced releasing of cytokines in microglia via targeting TLR2/MyD88/SNAP23 signaling. **a** Expression of SNAP23 and p-SNAP23 in microglia were measured by western blotting assay. **b** qRT-PCR was performed to detect expression of genes of IL-1β, IL-6 and TNF-α. **c** Concentration of IL-1β, IL-6 and TNF-α in microglia were detected using ELISA assay. **P < 0.01 contrasted with Ctrl group, ^##^*P* < 0.01 contrasted with LPS group, and ^$$^*P* < 0.01 compared with LPS + BTX-A group

To ensure whether BTX-A inhibits SNAP23 expression and phosphorylation and releasing of cytokines via repressing TLR2/MyD88 signaling, we constructed the overexpression system of TLR2, MyD88 and SNAP23, respectively. TLR2-, MyD88-, and SNAP23-overexpressed plasmid were successfully transfected into microglia (Additional file 2: Figure S2). In addition, the expression of TLR2, MyD88, and SNAP23 in microglia were notably upregulated by pcDNA-TLR2 (Additional file 3: Figure S4A), pcDNA-MyD88 (Additional file 3: Figure S4B), and pcDNA-SNAP23 (Additional file 3: Figure S4C), respectively. Our results demonstrated that the inhibition of BTX-A to SNAP23 expression and phosphorylation were partly reversed by overexpression of TLR2 and MyD88 (Fig. 4a). BTX-A inhibited SNAP23 phosphorylation in LPS-stimulated microglia via inhibition of TLR2/MyD88 signaling. Besides, the inhibitory effect of BTX-A on IL-1β, IL-6 and TNF-α mRNAs expression were partly reversed by TLR2, MyD88 and SNAP23 overexpression (Fig. 4b). The inhibition of BTX-A to IL-1β, IL-6 and TNF-α production were also rescued by overexpression of TLR2, MyD88 and SNAP23 (Fig. 4c). These results confirmed that BTX-A suppressed releasing of cytokines in LPS-stimulated microglia through repressing SNAP23 expression and phosphorylation by suppression of TLR2/MyD88 signaling.

### BTX-A inhibited SNAP23 phosphorylation via inactivation of IKKα/β

**Fig. 5:**
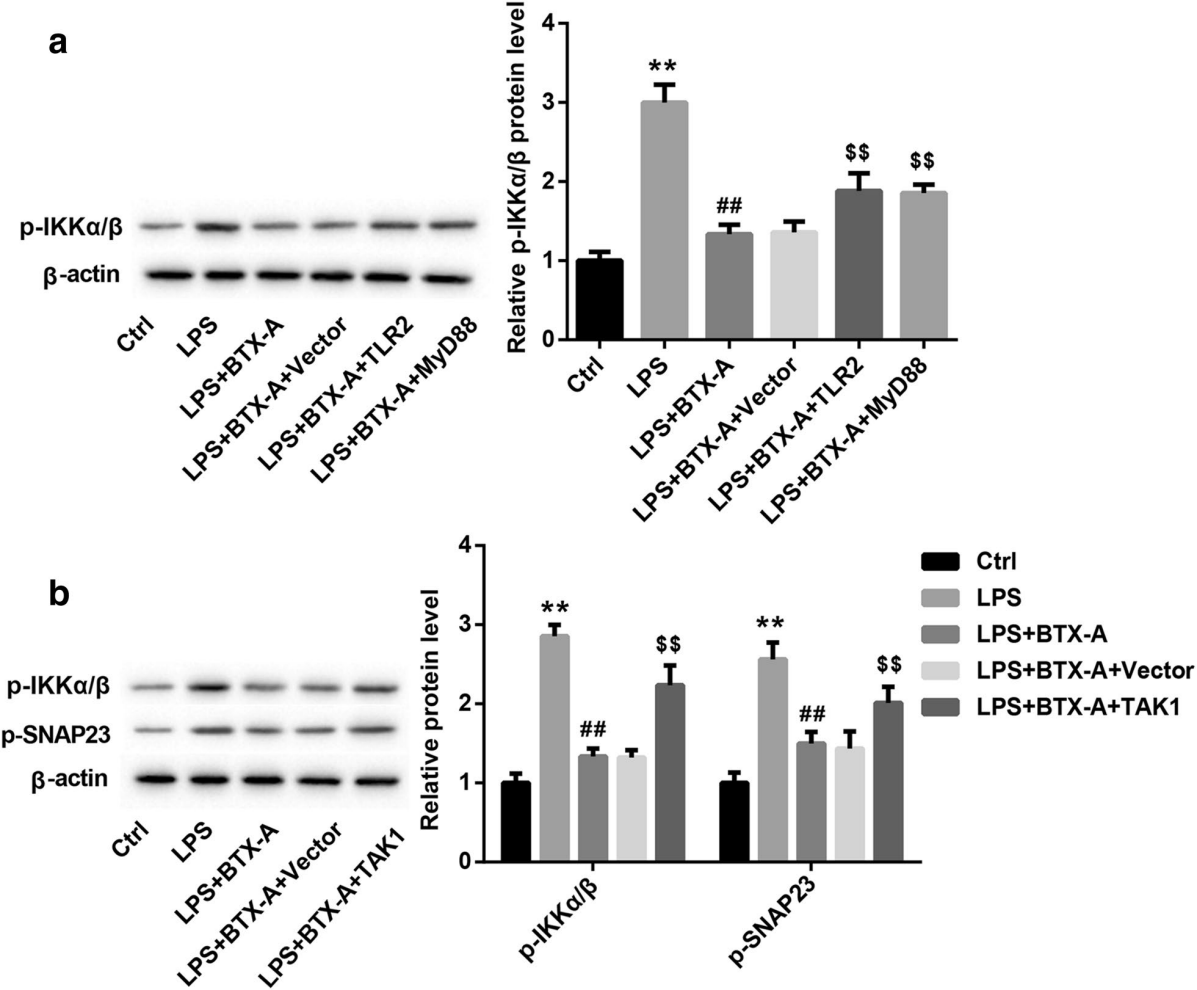
BTX-A inhibited IKKα/β activation and phosphorylation of SNAP23 via restraining TLR2/MyD88 signaling. **a** Expression of p-IKKα/β in microglias transfected with pcDNA-TLR2 or pcDNA-MyD88 following LPS and BTX-A was measured by western blotting assay. **b** Expression levels of p-IKKα/β and p-SNAP23 in microglias transfected with pcDNA-TAK1 following LPS and BTX were determined using western blotting assay. **P < 0.01 contrasted with Ctrl group, ^##^*P* < 0.01 contrasted with LPS group, and ^$$^*P* < 0.01 compared with LPS + BTX-A group

Furthermore, we found that phosphorylation level of IKKα/β was notably upregulated in LPS-stimulated microglia, and BTX-A could inhibit IKKα/β phosphorylation. However, overexpression of TLR2 and overexpression of MyD88 significantly declined the inhibitory effect of BTX-A on p-IKKα/β expression (Fig. 5a). Based on these findings, we proposed that BTX-A may be inhibit the phosphorylation of SNAP23 through suppression of the activation of IKKα/β. It was demonstrated that transforming growth factor-beta-activated kinase 1 (TAK1) induces phosphorylation of IKKα/β [12]. Here, we constructed the overexpression plasmid of TAK1. Our data displayed that pcDNA-TAK1 was successfully transfected into microglia (Additional file 2: Figure S2). The expression of TAK1 in microglia was obviously increased by pcDNA-TAK1 transfection (Additional file 3: Figure S4D). As shown in Fig. 5b, the inhibition of BTX-A to p-IKKα/β and p-SNAP23 expression were partly reversed by TAK1 overexpression. In summary, BTX-A suppressed the phosphorylation of SNAP23 through inactivation of IKKα/β by inhibition of TLR2/MyD88 signaling.

### BTX-A promoted SNAP23 ubiquitination via targeting NEDD4

**Fig. 6:**
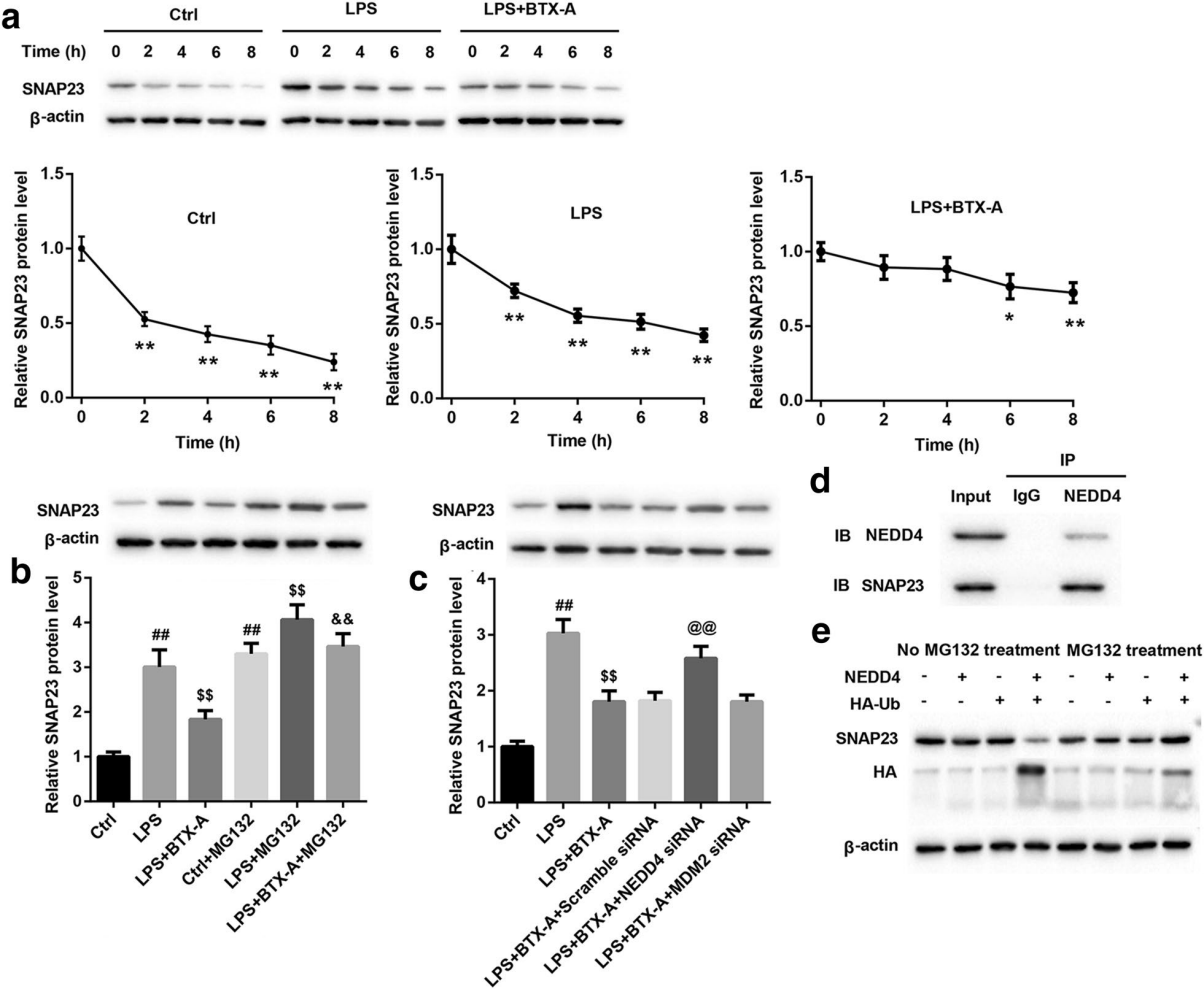
BTX-A promoted ubiquitin-degradation of SNAP23 by targeting NEDD4. **a** CHX was used to treat microglia following LPS and BTX-A. Expression of SNAP23 was measured by western blotting assay at 0, 2, 4, 6, 8 h later, respectively. The stabilization of SNAP23 was analyzed. **b** MG132 was used to inhibit ubiquitin-degradation of protein. Expression of SNAP23 was examined using western blotting assay. ^##^*P* < 0.01 compared with Ctrl group, ^$$^*P* < 0.01 contrasted with LPS group, and ^&&^*P* < 0.01 compared with LPS + BTX-A group. **c** Western blotting assay was performed to ensure expression of SNAP23 in microglia. ^##^*P* < 0.01 compared with Ctrl group, ^$$^*P* < 0.01 contrasted with LPS group, and ^@@^*P* < 0.01 compared with LPS + BTX-A group. **d** Co-IP assay was carried out to clarify the interaction between NEDD4 and SNAP23. IgG was served as control group. **e** HA-Ub and pcDNA-NEDD4 were co-transfected into HEK293T cells treated with or without MG132. Expression of SNAP23 and the level of SNAP23 ubiquitination were ensured using western blotting assay

To ensure whether BTX-A regulates the stabilization of SNAP23, microglias were treated with CHX, an inhibitor of protein synthesis, following LPS and BTX-A. At 0, 2, 4, 6, 8 h later for CHX induction, the expression of SNAP23 was examined. Our data showed that the synthesis of SNAP23 was inhibited by CHX in all groups (Ctrl group, LPS group and LPS + BTX-A group). Importantly, in Ctrl and LPS groups, expression of SNAP23 was obviously downregulated at 2 h later for CHX incubation. However, in LPS + BTX-A group, expression of SNAP23 was notably downregulated at 6 h for CHX induction (Fig. 6a). BTX-A delayed the occurrence time of downregulation of SNAP23. Next, microglia was treated with MG132, an inhibitor of proteasome, following LPS and BTX-A. Here, we revealed that the expression of SNAP23 was increased in microglias treated with LPS, and LPS and BTX-A by MG132 treatment (Fig. 6b). BTX-A inhibited SNAP23 expression via boosting its ubiquitin-degradation. Furthermore, we found that SNAP23 may be regulated by two E3 ubiquitin ligase: NEDD4 and MDM2. Hence, to clarify which one E3 ubiquitin ligase mediates the inhibition of BTX-A to SNAP23 expression, we transfected NEDD4 siRNA, MDM2 siRNA or scramble siRNA into microglias co-treated with LPS and BTX-A. NEDD4 siRNA, MDM2 siRNA, and scramble siRNA were successfully transfected into cells (Additional file 4: Figure S3). Meantime, the expression of NEDD4 and MDM2 were significantly reduced by the specific siRNA, respectively (Additional file 5: Figure S5A and S5B). Interestingly, western blotting assay results indicated that the inhibitory effect of BTX-A on expression of SNAP23 was reversed by NEDD4 decreasing, not MDM2 decreasing (Fig. 6c). In LPS and BTX-A co-treated microglia, the bound between NEDD4 and SNAP23 was proved using Co-IP assay (Fig. 6d). To clarify NEDD4 mediates the ubiquitination of SNAP23, we obtained HA-tagged ubiquitin (HA-Ub) and pcDNA-NEDD4, which were then transfected into HEK293T cells. At 48 h later, the cells were treat with or without MG132 for another 2 h. Our data demonstrated that the expression of SNAP23 in MG132-stimulated HEK293T cells co-transfected with pcDNA-NEDD4 and HA-Ub significantly higher than the cells untreated with MG132. The level of SNAP23 ubiquitination in MG132-treated cells higher than in MG132-untreated cells co-transfected with pcDNA-NEDD4 and HA-Ub (Fig. 6e). Taken together, we proved that BTX-A promoted ubiquitination of SNAP23 via targeting NEDD4.

### BTX-A facilitated NEDD4 expression via inhibition of TLR2/MyD88 signaling

**Fig. 7:**
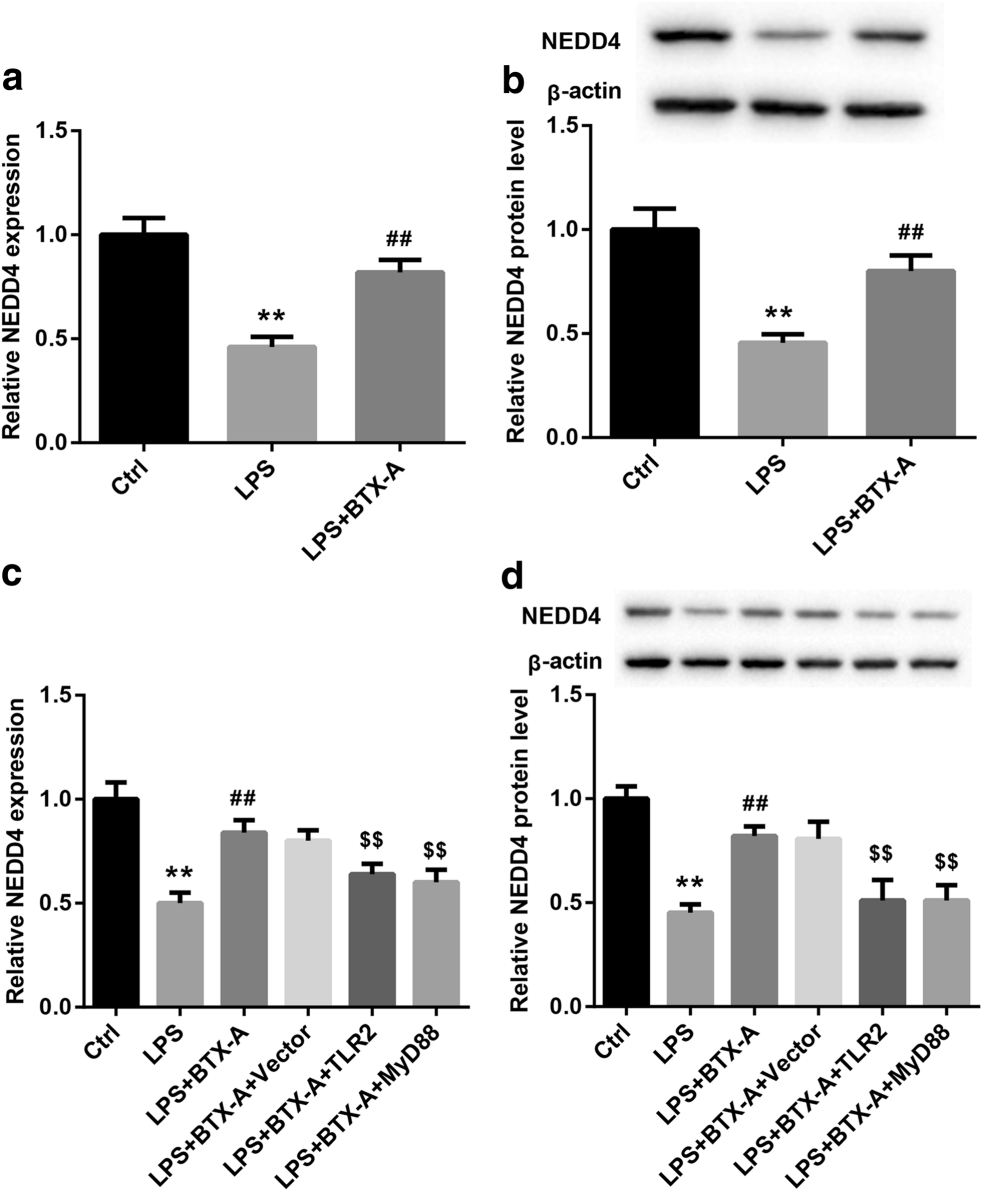
BTX-A upregulated NEDD4 via inhibiting TLR2/MyD88 signaling. **a** and **b** Expression of NEDD4 gene and protein were measured using qRT-PCR and western blotting assay, respectively. **c** and **d** qRT-PCR and western blotting assay were carried out to examine expression of NEDD4 gene and protein. **P < 0.01 contrasted with Ctrl group, ^##^*P* < 0.01 contrasted with LPS group, and ^$$^*P* < 0.01 compared with LPS + BTX-A group

Subsequently, to investigate whether TLR2/MyD88 signaling mediates the effect of BTX-A on NEDD4, following experiments were accomplished. Firstly, we ensured the influence of BTX-A to NEDD4 expression. Our data showed that expression of NEDD4 mRNA and protein in microglia were repressed by LPS, while were then partly reversed by BTX-A treatment (Fig. 7A and B). Next, pcDNA-TLR2, pcDNA-MyD88 or empty vector were transfected into microglia treated with LPS and BTX-A. As shown in Fig. 7c and d, the inhibition of BTX-A to NEDD4 mRNA and protein expression were partly attenuated by TLR2 and MyD88. Overall, our results proved that BTX-A accelerated the expression of NEDD4 in LPS-stimulated microglia through inhibition of TLR2/MyD88 signaling.

## Discussion

BTX-A is a neurotoxin released by Gram-positive anaerobic. BTX-A widely be utilized for muscle hyperactivity treatment because of its ability to suppress synaptic exocytosis [13]. Synaptic exocytosis can cause the relaxation of muscle. Contrasted with placebo, BTX-A could effectively attenuate pain intensity over 24 weeks in the patients with NP [14]. Moreover, Wei et al. also demonstrated that BTX-A has a better curative effect on trigeminal neuralgia and peripheral neuropathic pain when compared with placebo [15]. BTX-A maybe a potential therapy strategy in NP. However, the mechanism of action of BTX-A improves NP is not clear. During central nervous system (CNS), glial cells are an important participator in the process of neuronal homeostasis. Glial cells major consist of macroglia and microglia [16]. It was confirmed that microglia contributes to sensitization and maintenance in chronic pain through secretion of biologically active substances and pro-inflammatory factors [17]. A growing evidence indicated that spinal cord injury induced by NP can be effectively improved by the inactivation of microglia [18]. In this present study, we found that BTX-A reduced the production of pro-inflammatory factors in CCI-induced NP rats and LPS-stimulated microglia. Besides, we also found that BTX-A effectively attenuated pain in NP rats.

TLR2 is an important member of TLRs family. In CNS, TLR2 expressed in neurons, microglia and astrocytes, and closely associated with neuroninflammation pain [19]. It was indicated that the activation of TLR2 in microglia is important for an effective immune response against Gram-positive bacteria in CNS [20]. TLR2 signaling plays a crucial role in activation of spinal cord microglia induced by nerve injury. Recently, Yang et al. revealed that inhibition of TLR2/MyD88/NF-κB signaling in spinal microglia could attenuate pain in CCI-induced NP rats [21, 22]. Some researchers proposed that BTX-A promotes TLR2 expression in LPS-induced microglia [10]. But, our data found that BTX-A inhibited the expression of TLR2 and MyD88 in NP rats’ spinal cord tissues and LPS-stimulated microglia. We further demonstrated that BTX-A attenuated pain in NP rats, inhibited activation of microglia, and reduced production of cytokine factors through inhibition of TLR2/MyD88 signaling. In previous studies, Piotrowska et al. pointed out BTX-A restrains expression of SNAP23 in microglia [10]. Here, we also found that BTX-A reduced SNAP23 expression, and downregulated its phosphorylation via suppression of TLR2/MyD88 signaling. Subsequently, we indicated BTX-A impeded the production of pro-inflammatory factors through inhibition of SNAP23 expression by repressing TLR2/MyD88 signaling.

IκB kinase (IKK) complex plays a crucial role in transcriptional activation through regulation the phosphorylation of its target proteins. Some reliable evidences indicated that IKK mediates phosphorylation of SNAP23 [23, 24]. In this present study, we found that BTX-A reduced phosphorylation level of SNAP23 through inhibition of IKKα/β phosphorylation. Furthermore, we indicated that proteasome inhibitor reversed the inhibition of BTX-A to SNAP23 expression. Hence, we thought that BTX-A maybe decrease the expression of SNAP23 via enhancing its ubiquitination. The results of bioinformatic prediction showed that NEDD4 and MDM2, an E3 ubiquitin ligase, may mediate the ubiquitination of SNAP23. However, in our present study, NEDD4 downregulation, not MDM2 downregulation, reversed the effect of BTX-A on SNAP23 expression. Our data confirmed that BTX-A could facilitate the expression of NEDD4 via repressing TLR2/MyD88 signaling.

## Conclusions

Overall, our data demonstrated that BTX-A attenuated pain in NP rats through inhibition of pro-inflammatory factor production by reducing the level of SNAP23. Detailedly, on the one hand, BTX inhibited expression and phosphorylation of SNAP23 via repressing phosphorylation of IKKα/β. On the other hand, BTX-A reduced the level of SNAP23 through promotion the ubiquitination of SNAP23 via facilitating NEDD4 expression. Our study proved a novel action mechanism of BTX-A improve NP, and further confirmed the potential of BTX-A acts as treatment strategy for NP.

## Supplementary information

**Additional file 1: Figure S1.** Detection of the lentivirus infection. Spinal cord of rat were infected with lentivirus which carrying a GFP tag. Then, the infection efficiency in each group was examined under a microscope.

**Additional file 2: Figure S2.** Detection of the transfection efficiency of overexpression plasmid of genes. The GFP-marked overexpression plasmid of TLR2, MyD88, SNAP23, and TAK1 were constructed, and transfected into microglia. The transfection efficiency of these plasmids were examined under a microscope.

**Additional file 3: Figure S4.** Overexpression plasmid of TLR2, MyD88, SNAP23, and TAK1 promoted the expression of TLR2, MyD88, SNAP23, and TAK1, respectively. The overexpression plasmid of TLR2 (A), MyD88 (B), SNAP23 (C), and TAK1 (D) were transfected into microglia, and then the expression of these genes in protein level was measured by using western blotting assay. **P < 0.01 compared with Vector group.

**Additional file 4: Figure S3.** Detection of the transfection efficiency of siRNA. The GFP-marked siRNA of NEDD4 and MDM2, and scramble siRNA were constructed, and transfected into microglia. The transfection efficiency of these siRNAs were examined under a microscope.

**Additional file 5: Figure S5.** The expression of NEDD4 and MDM2 were reduced by the specific siRNA. (A) The siRNA of NEDD4 and scramble siRNA were transfected into microglia, and then the expression of NEDD4 was measured by using western blotting assay. (B) The siRNA of MDM2 and scramble siRNA were transfected into microglia, and then the expression of MDM2 was measured by using western blotting assay. **P < 0.01 compared with Scramble siRNA group.

## Data Availability

The datasets used and/or analysed during the current study are available from the corresponding author on reasonable request.

## Abbreviations

BTX-A: Botulinum toxin type A
CCI: Chronic compression injury
HA-Ub: HA-labeled Ubiquitin
IKKα: IκB kinase α
LPS: Lipopolysaccharide
MWT: Mechanical withdrawal threshold
NEDD4: Neural precursor cell-expressed developmentally downregulated gene 4
NP: Neuropathic pain
SNAP23: Synaptosome associated protein 23
TLR2: Toll-like receptor 2
TWL: Thermal withdrawal latency
