# Supplementary figures and images for: Botulinum toxin type A alleviates neuropathic pain and suppresses inflammatory cytokines release from microglia by targeting TLR2/MyD88 and SNAP23
Source: Cell Biosci. 2020 Dec 9;10:141. doi: 10.1186/s13578-020-00501-4 (PMC7724852; doi:10.1186/s13578-020-00501-4)

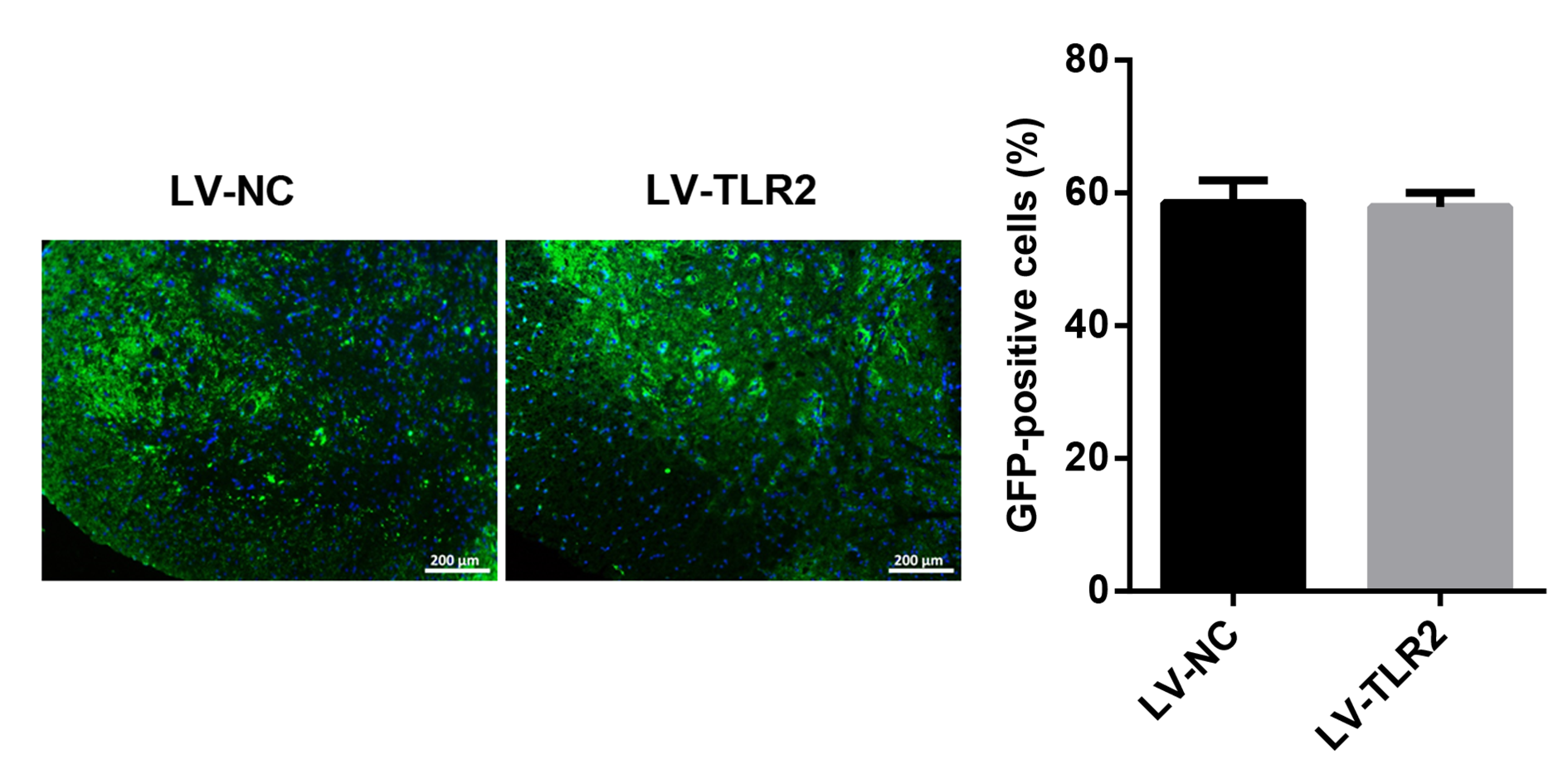

Supplement: Supplementary file 1 — Additional file 1: Figure S1. Detection of the lentivirus infection. Spinal cord of rat were infected with lentivirus which carrying a GFP tag. Then, the infection efficiency in each group was examined under a microscope. [file 13578_2020_501_MOESM1_ESM.tif]

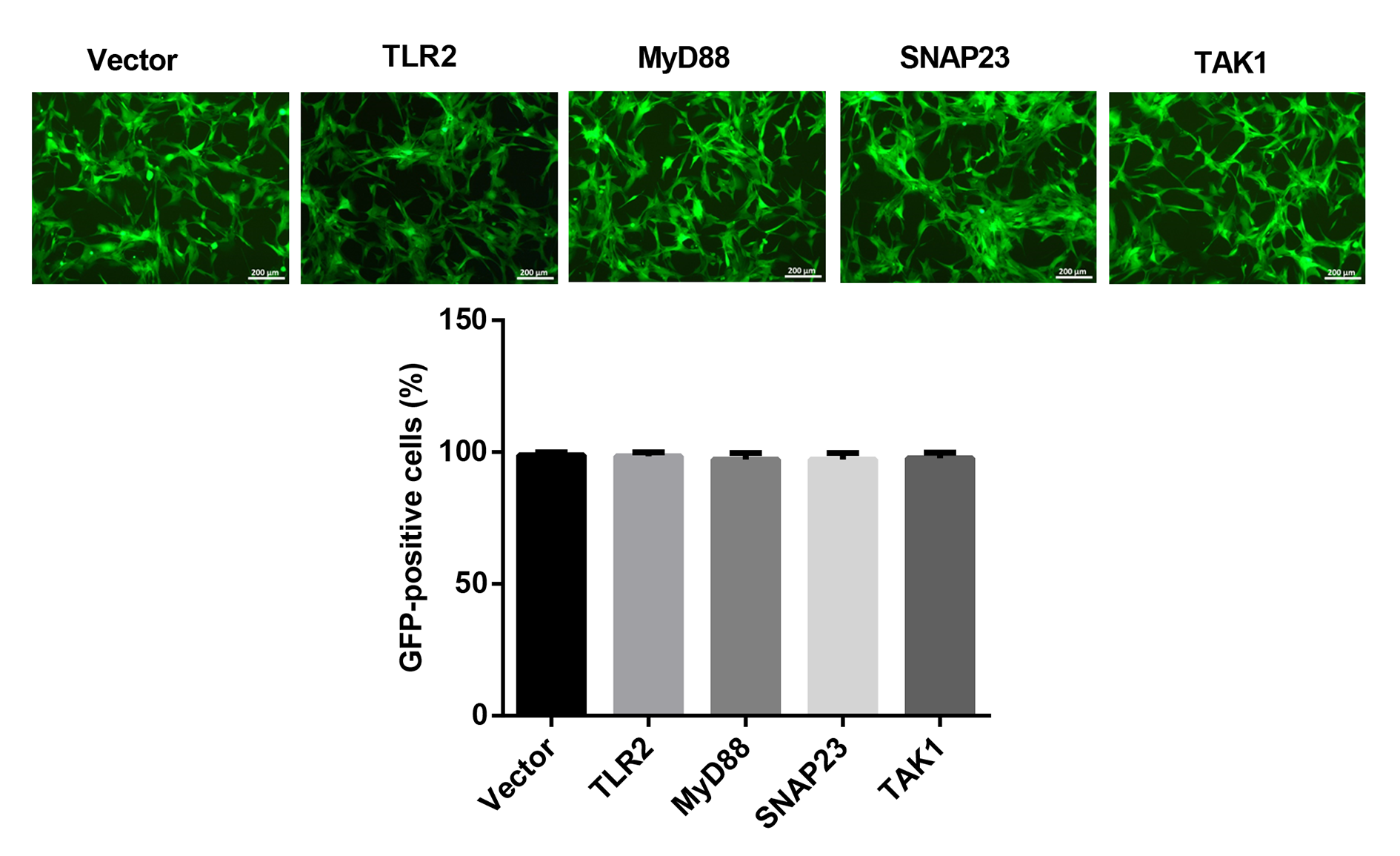

Supplement: Supplementary file 2 — Additional file 2: Figure S2. Detection of the transfection efficiency of overexpression plasmid of genes. The GFP-marked overexpression plasmid of TLR2, MyD88, SNAP23, and TAK1 were constructed, and transfected into microglia. The transfection efficiency of these plasmids were examined under a microscope. [file 13578_2020_501_MOESM2_ESM.tif]

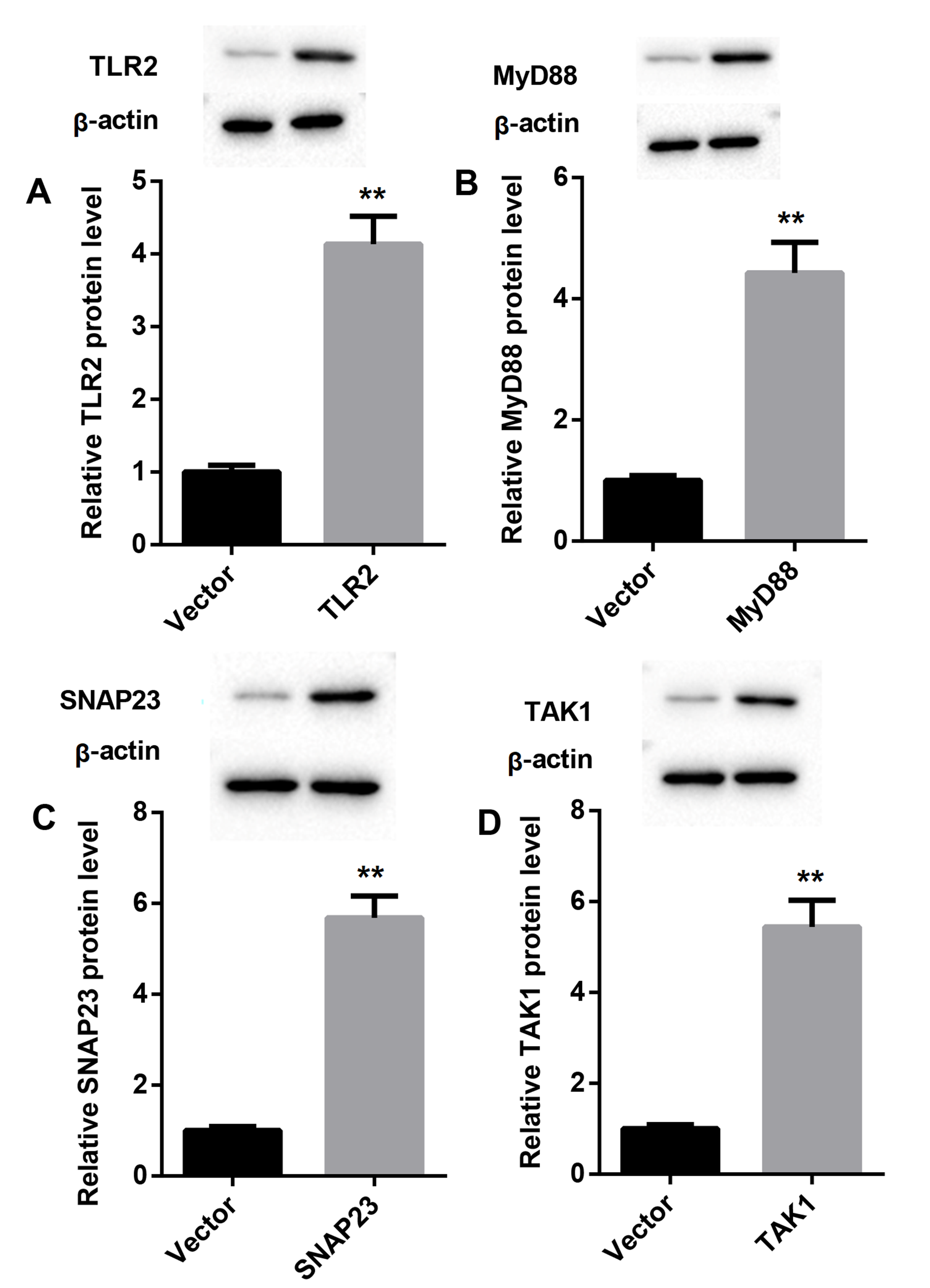

Supplement: Supplementary file 3 — Additional file 3: Figure S4. Overexpression plasmid of TLR2, MyD88, SNAP23, and TAK1 promoted the expression of TLR2, MyD88, SNAP23, and TAK1, respectively. The overexpression plasmid of TLR2 (A), MyD88 (B), SNAP23 (C), and TAK1 (D) were transfected into microglia, and then the expression of these genes in protein level was measured by using western blotting assay. **P < 0.01 compared with Vector group. [file 13578_2020_501_MOESM3_ESM.tif]

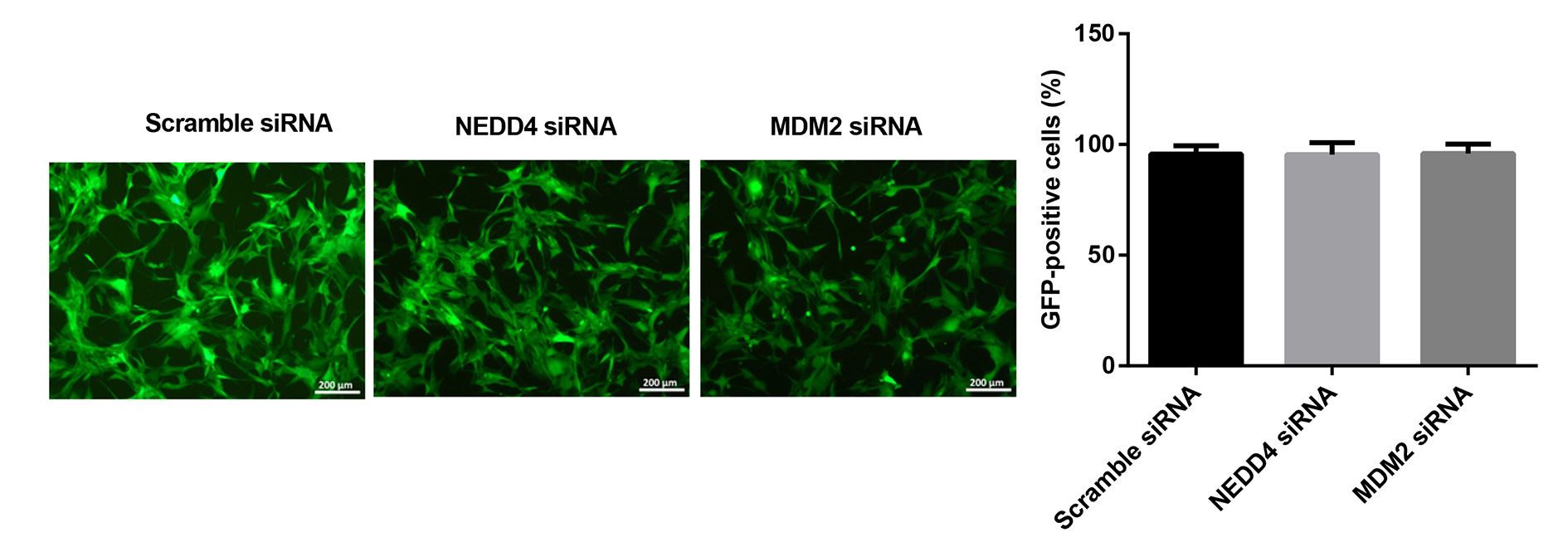

Supplement: Supplementary file 4 — Additional file 4: Figure S3. Detection of the transfection efficiency of siRNA. The GFP-marked siRNA of NEDD4 and MDM2, and scramble siRNA were constructed, and transfected into microglia. The transfection efficiency of these siRNAs were examined under a microscope. [file 13578_2020_501_MOESM4_ESM.tif]

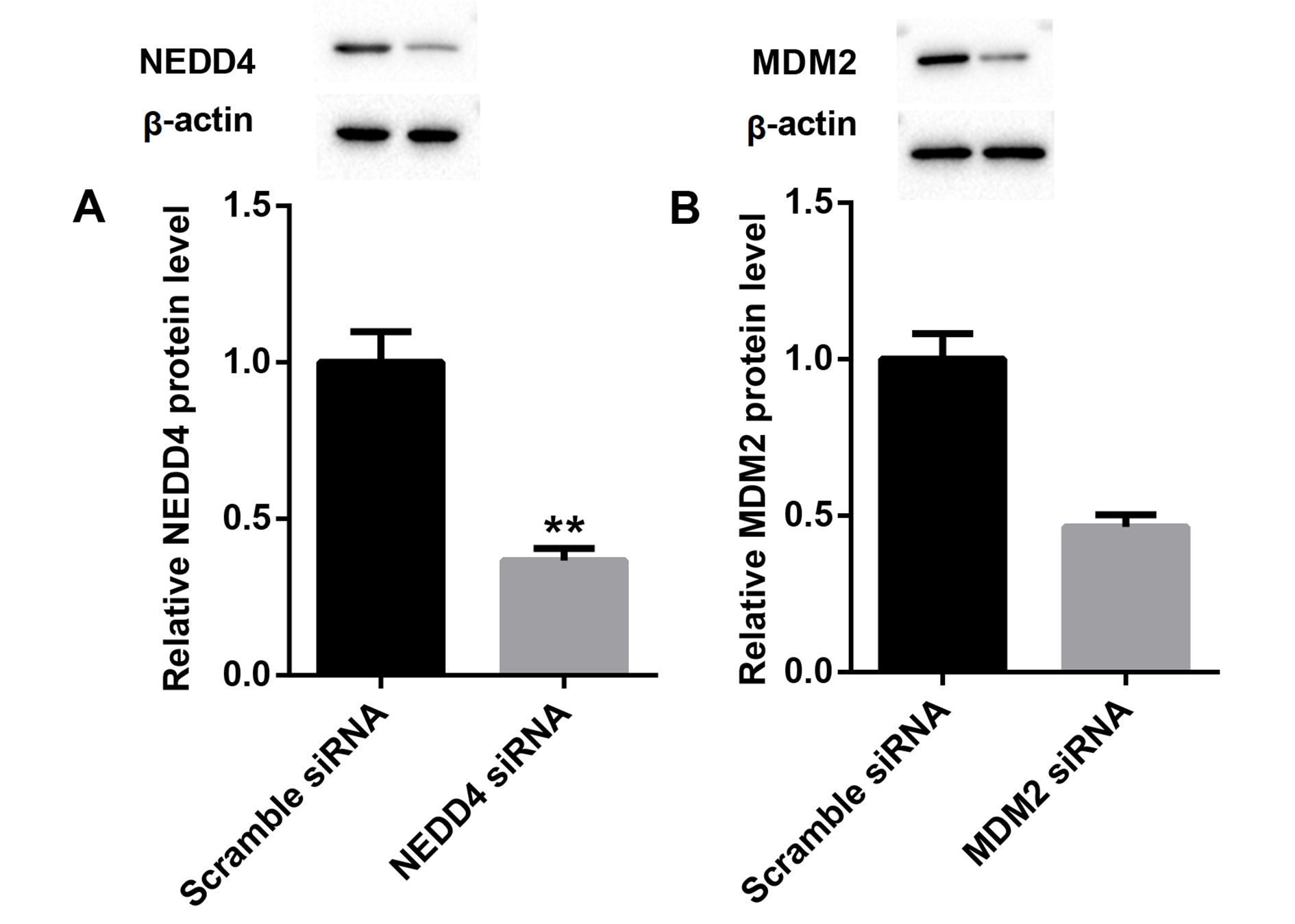

Supplement: Supplementary file 5 — Additional file 5: Figure S5. The expression of NEDD4 and MDM2 were reduced by the specific siRNA. (A) The siRNA of NEDD4 and scramble siRNA were transfected into microglia, and then the expression of NEDD4 was measured by using western blotting assay. (B) The siRNA of MDM2 and scramble siRNA were transfected into microglia, and then the expression of MDM2 was measured by using western blotting assay. **P < 0.01 compared with Scramble siRNA group. [file 13578_2020_501_MOESM5_ESM.tif]
